# Context-sensitive holistic care of women with one previous Caesarean section

**DOI:** 10.4102/safp.v66i1.5879

**Published:** 2024-01-30

**Authors:** Adam K. Asghar, Evashnee Naidoo

**Affiliations:** 1 Department of Family Medicine, KwaZulu-Natal Department of Health, Durban, South Africa; 2 Mama Earth Midwifery Care Centre, Durban, South Africa

**Keywords:** vaginal birth after Caesarean, obstetrics, labour and delivery, holistic medicine, South Africa, patient-centred care

## Abstract

This article’s emphasis is on the holistic care of women who are assessed as suitable for and amenable to vaginal birth after Caesarean section (VBAC) in the South African state health sector context. It is beyond its scope to deal with the minutiae of VBAC conduct, operative conduct of repeat Caesarean section (CS), or management of uterine rupture. It is also beyond the scope of the article to reflect on practices, which are accepted in other healthcare contexts. The intention is not to promote VBAC over elective repeat CS, but rather to assist healthcare workers with providing high-quality holistic care. The goal is that women with previous CS are given access to the mode of delivery, which is safest for them and their fetus, while minimising adverse psychological effects of previous and future negative birth experiences.

## Introduction

Pregnancy and delivery are natural processes, which do not always benefit from interventions. Over-medicalisation is a point of contention in the obstetric and midwifery community of practice.^1^ However, it remains that on an individual level, positive pregnancy and birth experiences aligned with a woman’s needs and expectations should be facilitated.^2^ From a public health perspective, healthcare workers (HCWs) should be mindful of overburdening health systems with unnecessary interventions and their sequelae. This is especially true of Caesarean section (CS), a potentially life-saving but life-threatening intervention, the latter outcome related not only to its specific complications (e.g. haemorrhage, sepsis, venous thromboembolism and anaesthetic issues) but also to the pathology making it a necessity (e.g. eclampsia).

Recent South African (SA) data show that although the CS rate is static at around 28%, the CS case fatality rate has risen.^3^ This is a stark reminder of the need to exercise caution when offering CS; health systems initiatives have been developed to address this, namely Emergency Obstetric Simulation Training (EOST) modules related to cardiotocograph (CTG) interpretation, labour monitoring, and assisted delivery skills, and facility-based CS auditing.^3^

One of the biggest contributors to the likelihood of having a CS in the index pregnancy is having had a previous CS. This ‘snowball effect’ that starts with a woman’s first CS, includes an accrual of medical risk with each subsequent CS (intra-abdominal adhesions, placenta praevia and accreta spectrum, surgical injury, hysterectomy, blood transfusions).^4^

All of this should be balanced against the risks associated with a vaginal birth after Caesarean section (VBAC) – decades ago an unthinkable option: ‘Once a Caesarean, always a Caesarean’.^5^ According to present day norms, for someone who has had one previous CS, there are three possible outcomes in the index pregnancy:

- successful VBAC
- elective repeat Caesarean section (ERCS)
- unsuccessful VBAC leading to an emergency CS.

The last option confers the potential for maternal disappointment and the highest medical risk as follows:

- maternal: prolonged hospital stay, higher incidences of wound infection/fever/haemorrhage/urinary tract infection/death
- fetal/neonatal: lower Apgar scores, hypoxia, admission to neonatal intensive care unit, death.

Caesarean section without medical indication is not available in the SA state health sector. However, in the context of one previous CS, because the possibility of a repeat CS is close to 40%, a woman’s preference for mode of delivery (MoD) is considered. This preference should be based on the woman having access to unbiased evidence-based information regarding her birth options, free of coercion, with sufficient time to consider these options.^6^ The overall aim of HCWs caring for a woman who has had one CS, should be to support her right to experience a suitable MoD of her choice.

## Vaginal birth after Caesarean section versus elective repeat Caesarean section

Healthcare workers should be able to guide a woman through the information presented in Table 1 based on her individual risk profile and inherent preferences, validating correct knowledge, and correcting misperceptions.^7^

**TABLE 1:** Pros and cons of the modes of delivery available to women who have delivered by one Caesarean section in the past.

Associated features	Vaginal birth after Caesarean section	Elective repeat Caesarean section
Advantages	Avoid risk of CS Less post-partum pain Shorter hospital stay Possible greater psychological satisfaction Reduced cost for patient and/or state Better breastfeeding outcomes Microbiome transfer	Convenience of planning Safer than emergency CS Lower risk of uterine rupture/perineal trauma than VBAC Tubal ligation can be performed at same sitting Slight reduction in mother-to-child transmission (unsuppressed HIV)
Disadvantages	Labour pain Risk of uterine rupture (< 1%) with maternal/neonatal sequelae Additional risk of emergency CS in case of an unsuccessful VBAC Increased neonatal morbidity risk	Surgical/anaesthetic risk Increased chance of neonatal admission Loss of MoD flexibility (will need ERCS in future pregnancies)

*Source*: Adapted from Moodley J, editor. A monograph on Caesarean section. Pretoria: National Department of Health, Republic of South Africa; 2013

CS, Caesarean section; VBAC, vaginal birth after Caesarean section; HIV, human immunodeficiency virus; MoD, mode of delivery; ERCS, elective repeat Caesarean section.

The process of planning an appropriate MoD after one previous CS starts immediately after the primary CS, when the HCW can offer the woman a narrative of the indication of the primary CS, and of the impact that her birth experience will have on future pregnancies.^8^ Such a debriefing allows HCWs to identify women who may require additional postnatal support, which includes vulnerable groups such as teenagers and those who have had a disempowering birth experience.^9^ In addition, the immediate post-partum period offers a chance to promote planning a future pregnancy at an appropriate inter-delivery interval, by offering long-acting reversible contraceptives, and encouraging early future antenatal booking.

In the SA state health sector, once a woman falls pregnant after one CS and ‘books’ antenatal care at a primary healthcare (PHC) site, in addition to gaining access to the standard antenatal care package (which should include an early obstetric ultrasound to confirm gestational age and placentation), she should be referred to see a doctor shortly after booking so that the events around the previous CS can be reviewed and documented, and MoD planning can begin.^10^

While patient-centredness (one of the core features of family medicine) should characterise every consultation, considering staff shortages in the SA state health sector, it is important to identify patients who require a more in-depth individualised approach because of unresolved birth trauma, which can have a negative impact on a woman’s current pregnancy, birth, postnatal and parenting experiences.^11,12^ The nature of a patient’s responses to questions around previous birth experiences and expectations for the current pregnancy can provide insights, which will serve as foundations of holistic care planning.

Individual counselling can be enhanced with patient information leaflets/videos, and peer support groups.^13^ If no other risk factors are identified, most women with one previous CS will be eligible to continue antenatal care at their PHC clinic, returning to a doctor-led ‘high-risk’ clinic at 36 weeks’ gestational age.

## Suitability versus amenability

The authors’ personal experience is that HCWs often fall into the trap of determining a patient’s MoD based on her amenability only. The Caesarean Section Monograph (written by the National Committee on Confidential Enquiries into Maternal Deaths as an intervention to address rising CS case-fatality rates observed over a decade ago) guides HCWs to assess suitability first, which involves determining whether it is safe for a patient to undertake a VBAC attempt, and whether this attempt is likely to be successful (Table 2).^7,14,15^ The authors suggest a hybrid approach, whereby the patient’s ideas are invited first, which may bring up questions like ‘Will I be able to deliver naturally?’. Such a question should be validated by the HCW (so that underlying concerns can be unearthed, and the patient leaves the consultation feeling heard and assisted) and responded to by stating that it will be answerable after the information-gathering and physical examination phases of the consultation have been completed.

**TABLE 2:** Criteria for assessing suitability for vaginal birth after Caesarean section.

Absolute contraindications	Relative predictors of unsuccessful attempt
Lack of patient consent More than one previous CS Previous non-lower segment CS (e.g. classical/hysterotomy/de Lee/J-shaped) or suspicion thereof (e.g. history of previous CS performed at < 32 weeks’ gestational age; patient reports that she was counselled against pursuing VBAC during a previous pregnancy; supra-umbilical midline skin incision for a previous CS in a patient who is unable to confirm that a lower segment CS was performed) Previous surgery on uterine body/fundus (e.g. myomectomy, laparotomy for cornual ectopic pregnancy) Previous ruptured/perforated uterus Contracted pelvis Conditions where CS is indicated (e.g. malpresentation, multiple pregnancies, placenta praevia) Other conditions (e.g. hypertension, fetal growth restriction) where expedited delivery is required Maternal booking BMI > 40 kg/m^2^	An inter-delivery interval of < 18 months (related to CS i.e. ‘fresh scar’) or > 10 years (related to the last delivery, irrespective of mode) Maternal age of ≥ 40 years Post-dates gestational age (fetal head harder and less likely to ‘mould’ in the pelvis) – mechanical induction can be offered to those women who wish to avoid ERCS, and do not have multiple additional relative predictors of an unsuccessful attempt Previous CS performed as an emergency: specifically for cephalopelvic disproportion as an indication and if estimated fetal weight of index pregnancy is bigger than birthweight at first CS No prior normal vaginal delivery Booking BMI > 30 kg/m^2^ Multiple other risk factors Unfavourable cervix Previous perinatal death (neonatal demise < 7 days age or stillbirth) Symphysial fundal height ≥ 40 cm (suggestive of estimated fetal weight > 3.5 kg)

*Source:* Adapted from Green-Thompson RR. Protocol for patients with one previous caesarean section undergoing vaginal birth after caesarean section (VBAC). Durban: King Edward VIII Hospital; 2021

CS, Caesarean section; BMI, body mass index; VBAC, vaginal birth after Caesarean section; ERCS, elective repeat Caesarean section.

Once suitability has been thoroughly assessed, amenability can be revisited. There are a variety of factors that women in an SA state health sector setting with one previous CS have been shown to use in determining their preferred MoD. These include the fear of labour pain (referred to as tocophobia when at an unreasonable level), wanting to feel ‘pushing’, fear of CS pain, the HCW’s recommendation, and recovery times. It should be noticed that both MoDs invoke fear of pain, which serves as a reminder for HCWs to prioritise the timely provision of labour/post-partum analgesia and also communicate to patients the commitment to addressing pain. Neglected pain management in a previous birth experience can be a powerful driver, together with word of mouth, towards an expressed choice of ERCS. In addition to explicitly stated reasons for a preferred MoD, demographic features, such as cultural norms and relationship status have been shown to be associated with a preferred MoD.^16,17^

## Intrapartum care

The intrapartum care of VBAC should occur at a site which has access to safe (according to the national Minimum Standards) emergency CS at any time of the day, with emergency blood available on site.^18^ Key steps in intrapartum care include:

1. Priority assessment (on arrival, or at the onset of suspected labour) by a senior HCW, with a review of the pre-existing MoD plan.
2. If in labour (regardless of the phase) or in suspected labour (until proven otherwise):
  - Admission to the labour ward
  - Establishing intravenous access while supporting oral intake, to prevent exhaustion
  - Respectful obstetric care, preserving the parturient’s dignity and autonomy, and including clear communication, pain management (non-pharmacological and pharmacological), and promotion of access to a birth companion.
  - Careful monitoring for maternal and fetal indicators of imminent and suspected uterine rupture (which warrant an emergency CS/laparotomy).
  - Strict partogram use, responding to prolonged labour (in any phase) by recommending an emergency CS.
  - Avoidance of uterotonic agents.

The Caesarean Section Monograph recommends continuous CTG monitoring, but highlights that VBAC may be conducted without it, if the monitoring of both maternal contractions and fetal heart is performed and documented according to a strict schedule.^7^ It is found that continuous CTG monitoring can jeopardise the success of a vaginal delivery, as it can limit maternal positions during labour and delivery (either through affecting comfort or through compromising the diameter of the pelvic outlet and the assistance of gravity).^19^ If staffing levels allow (i.e. continuous one-to-one care is possible), intermittent monitoring can be pursued, in an effort to maximise the success of a VBAC attempt, but never at the cost of safety – uterine rupture is more likely to occur if there are insufficient staff to monitor the patient adequately.^20^

If a VBAC attempt appears to be not proceeding smoothly (either in terms of progress of labour despite rupture of membranes or because of suspicious/pathological features of materno-fetal monitoring), this should be sensitively communicated to the parturient, avoiding use of words such as ‘failed’, because of the psychological consequences of being informed that the labour failed. They should be counselled about the risks of pursuing VBAC further and have an emergency CS recommended. This is especially true if non-modifiable predictors of an unsuccessful attempt are present.

Following a successful VBAC, post-partum care should not follow the standard schedule for a normal vaginal delivery, with attention paid to markers of a late uterine rupture. Whatever the MoD achieved, screening for a negative birth experience should be conducted; unresolved guilt associated with labour not having gone according to plan, can cause not only direct psychological stress to the post-partum woman but also confer risk to the infant, in terms of care and attachment.

## Clinical governance

In addition to routinely running EOST drills, and auditing CSs, facilities should reflect on their VBAC performance, aiming to achieve a success rate of > 50%. Vaginal birth after Caesarean section success rate is calculated by dividing the number of VBACs completed with no adverse maternal/neonatal events by the total number of VBACs attempted (i.e. patients who were not suitable for or amenable to a VBAC are excluded). Poor performance should guide the facility to review antenatal/intrapartum patient selection practices, and the quality of intrapartum care.

## Conclusion

A pregnant woman who has had a previous CS is always a high-risk patient, not only from an obstetric perspective, but often from an additional psychological perspective. Antenatal, intrapartum and post-partum care plans for these patients should be aligned with the risk, such that women are offered the opportunity to give birth safely and ideally in a manner that aligns with their values and represents a positive and empowering experience. This requires the family physician to offer holistic care, which is in alignment with the global priority for all obstetric care to be characterised by respect of the pregnant and labouring woman.
